# Healthcare equity analysis: applying the Tanahashi model of health service coverage to community health systems following devolution in Kenya

**DOI:** 10.1186/s12939-019-0967-5

**Published:** 2019-05-07

**Authors:** Rosalind McCollum, Miriam Taegtmeyer, Lilian Otiso, Maryline Mireku, Nelly Muturi, Tim Martineau, Sally Theobald

**Affiliations:** 1 Department of International Public Health, Liverpool School of Tropical Medicine, Pembroke Place, Liverpool, UK; 2 LVCT Health, Nairobi, Kenya

**Keywords:** Devolution, Kenya, Tanahashi, Equity, Health services

## Abstract

**Background:**

Universal health coverage (UHC) is growing as a national political priority, within the context of recently devolved decision-making processes in Kenya. Increasingly voices within these discussions are highlighting the need for actions towards UHC to focus on quality of services, as well as improving coverage through expansion of national health insurance fund (NHIF) enrolment. Improving health equity is one of the most frequently described objectives for devolution of health services. Previous studies, however, highlight the complexity and unpredictability of devolution processes, potentially contributing to widening rather than reducing disparities. Our study applied Tanahashi’s equity model (according to availability, accessibility, acceptability, contact with and quality) to review perceived equity of health services by actors across the health system and at community level, following changes to the priority-setting process at sub-national levels post devolution in Kenya.

**Methods:**

We carried out a qualitative study between March 2015 and April 2016, involving 269 key informant and in-depth interviews from different levels of the health system in ten counties and 14 focus group discussions with community members in two of these counties. Qualitative data were analysed using the framework approach.

**Results:**

Our findings reveal that devolution in Kenya has focused on improving the supply side of health services, by expanding the availability, geographic and financial accessibility of health services across many counties. However, there has been limited emphasis and investment in promoting the demand side, including restricted efforts to promote acceptability or use of services. Respondents perceived that the quality of health services has typically been neglected within priority-setting to date.

**Conclusions:**

If Kenya is to achieve universal health coverage for all citizens, then county governments must address all aspects of equity, including quality. Through application of the Tanahashi framework, we find that community health services can play a crucial role towards achieving health equity.

## Introduction

Health equity and universal health coverage (UHC) are fundamentally about fairness and justice [1, 2]. Kenya has long described the importance of promoting health equity within policy documents and has made considerable progress in reducing mortality rates among children and improving coverage of health services [3]. More recently the attainment of UHC has been promoted as one of the four central pillars taken up by the president within the current government’s agenda [4]. Yet wide disparities in health outcomes and uptake of services persist, disadvantaging those most vulnerable [3]. In part as a response to citizen’s frustrations with the wide inequities between regions, Kenya devolved services (including health) in 2013. We know from global experience that while devolution brings with it expectations for improved equity, in practice it is a complex process, and outcomes can be unpredictable, potentially widening, rather than reducing, disparities [5–9]. Within Kenya, early study indicates that opportunities for local prioritisation and community involvement for equity in resources allocation, have not yet been harnessed [10].

Systematic disparities in access and use of healthcare services, and/or equity in health financing contribute to inequities in healthcare. While equity is implicit in universal health coverage (UHC), there is still a risk that poorer, less advantaged groups may be left behind, unless health systems maintain an adequate focus on the measurement of equity [11–13]. There is the need to consider whether UHC policies close, rather than widen disparities in use of health services and health outcomes, and whether the processes for planning and monitoring are implemented in a pro-equity manner [14].

UHC should provide “access to key promotive, preventive, curative and rehabilitative health interventions for all at an affordable cost, thereby achieving equity in access” [15]. A simple classification of services as high, medium and low priority^1^ is recommended, with countries not yet having universal coverage for all high-priority services recommended to first expand those, waiting to expand low or medium priority services until there is already near universal coverage for all high-priority services [16]. Although lack of agreement over what this means can create a barrier to decision-makers.

Evidence has consistently shown that disadvantaged groups have poorer survival chances and lower use of facility-based services [2, 4, 5]. Therefore, in order to “to achieve universal health coverage, health systems will have to reach into every community, including the poorest and hardest to access” (p.847 [17]). Within Kenya this has been interpreted as relating to improving access to national health insurance fund (NHIF), but for true UHC is to be achieved, it will require “the provision of needed, and *good quality health services* to the entire population, without the risk of financial ruin” (p.1175 [13]). Community health services have an important interface role to play in attaining UHC by involving and empowering of communities to change health-related beliefs, behaviours and improve access and uptake of health services [18].

In order to fully appreciate the impact of health reforms (such as devolution) on equity and universal health coverage it is helpful to consider Tanahashi’s concept of health service coverage (see Table 1), which emphasises quality and effectiveness when determining effective coverage [19]. Tanahashi’s model considers five elements necessary for effective coverage. At each coverage level, various factors within the health system work together, interacting to influence who has access to services, always with an awareness that there is the potential to lose people from the care seeking pathway at each stage. Tanahashi’s model is now relatively old, but there has been renewed interest in using it as a way to deconstruct aspects of equity as part of district health systems strengthening as part of the pathway to UHC [14]. While Tanahashi’s model has widely been used for analysis at national level, to date it has had more limited application at sub-national levels [20], particularly, after the introduction of devolution reforms.

**Table 1:** Mapping supply and demand determinants with Tanahashi levels of coverage

Supply and Demand	Tanahashi levels of coverage
Supply side determinants of the health system (those aspects of the health system which relate to the production of healthcare).	• Availability coverage – The availability of resources such as health workers, health facilities, drugs determines the extent to which a service can be provided**.** • Accessibility coverage – Defines the population who can use or access the service. A service has to be geographically accessible, located within reasonable reach of people who need it and financially affordable.
Demand side determinants (those aspects operating at individual, household or community level, which influence the ability of an individual to identify illness, and willingness to seek and use appropriate health care).	• Acceptability coverage – This domain defines the people who can access the service, are willing to use it and finds it acceptable for example in terms of costs, waiting time, beliefs. • Contact coverage – These are people who have been in contact with the service provider and have utilised the service. • Effectiveness coverage – The proportion of the population in need of an intervention that receive an effective intervention.

Sources (Frenz and Vega 2010; World Health Organisation (WHO) 2010; Tanahashi 1978; Ensor and Cooper 2004; Henriksson et al. 2017)

Following devolution reforms in Kenya, two levels of government are formally recognised – national level and 47 county governments. A range of sources of revenue are available to county governments to fund devolved functions (including health) (see Table 2). The changing process for setting priorities following devolution, provides opportunity to promote equity through UHC. Bringing decision-making closer to citizens allows for greater responsiveness to varied citizen demands, increased inclusivity of marginalised groups within decision-making, along with opportunity for intersectoral collaboration across departments to identify actions which both increase use of services among disadvantaged populations and address social determinants of health [8]. Despite many positive expectations, evidence in fact suggests that ‘decentralisation has done little to improve the quantity, quality or equity of public services in the (Sub-Saharan Africa) region’ (p.21 [21]). It is therefore crucial to evaluate stakeholder perceptions surrounding whether the anticipated benefits, in fact come to pass. We sought to explore three questions: What does equity mean to those involved with setting priorities? What are the implications of devolution for the dimensions of equity identified by Tanahashi? How can health equity be improved?

**Table 2:** Funding sources in Kenya following devolution.

County governments receive funding from three possible sources	
1) transfers from central government which comprise an equitable share allocated to all the 47 counties from national general revenue collections using a revenue allocation formula, conditional grants ring fenced for specific functions, and an equalization fund for the 14 previously marginalized counties	
2) locally generated revenue	
3) donor funding The county government authorities now have responsibility for budget allocation of these funds and annual planning. These county authorities also hold responsibility for developing the five-year county integrated development plan (CIDP) and five-year county strategic plan for health; health service delivery for level one to three services (community, primary and county referral services); recruitment and management of health workers and coordination of partners.	

Source: (Commission on revenue allocation 2016; Overseas Development Institute 2016; National Council for Law Reporting with the Authority of the Attorney-General 2010)

## Methods

### Data collection

We used a qualitative research methodology to explore inductively, the implications of devolution for equity, through generation of rich data by seeking to understand what equity means for those involved with decision-making and ‘how’ equity can be improved [22, 23]. This methodology gives “due emphasis to the meanings, experiences, and views of all the participants” (p. 43 [22]), to develop possible explanations and theories surrounding the implications of devolution for equity at multiple levels [24].

We carried out interviews with 269 individuals and 14 focus group discussions with an additional 146 participants, between March 2015 – April 2016 (see Table 4 in McCollum et al. (2018) [8]). Fourteen national level key informants were selected purposively. National, county and some health worker level interviews were carried out by the first author in English (a non-Kenyan national, trained qualitative researcher). 120 county level decision-makers were interviewed across the ten diverse study counties. Counties were selected to include representation of a range of poverty levels, geographic settings, cultural and social demography and health service coverage levels within the country (see Table 3 [8]). County level decision-makers were purposively selected to include politicians involved with decision-making for health, county treasury staff involved with budget guidance, gender and children’s office representatives and technical decision makers for health including members of the county health management team. Interviews with 49 health workers from sub-county, health facility and community levels were carried out in three of the ten counties, selected to include counties which aligned with REACHOUT^2^ consortium data collection and which included representation for urban, rural agrarian and rural pastoralist settings. 86 interviews with close-to-community (CTC) providers, their supervisors and community members and 14 focus group discussions were carried out with community members from two out of the three counties. This data was collected by Kenyan national researchers, trained in qualitative research as part of an ongoing REACHOUT CTC provider quality improvement study in two counties (urban and rural agrarian). We used topic guides to explore equity implications of health decisions made following priority-setting for health at county level following devolution. The topic guides were developed through an iterative process following informal discussions with national key informants, discussion between colleagues and a period of reflection and revision after data collection in one county to ensure questions elicited the responses sought.

**Table 3:** Key indices for study counties

County	Marginalised^3^/ not marginalised	Poverty incidence (Headcount ratio)(Kenya National Bureau of Statistics 2014)	Rural/urban	Province	Live births in previous 5 years % delivered by skilled provider(Kenya National Bureau of Statistics et al. 2014)	% children age 12–23 months who are fully vaccinated(Kenya National Bureau of Statistics et al. 2014)
Homa Bay	Not marginalised	48.4%	Rural agrarian	Nyanza	60.4%	53.7%
Kajiado	Not marginalised	38.0%	Rural nomadic	Rift Valley	63.2%	48.9%
Kitui^b^	Not marginalised	60.4%	Rural agrarian	Eastern	46.2%	52.7%
Kwale	Marginalised	70.7%	Rural agrarian	Coast	50.1%	82.0%
Marsabit^a^	Marginalised	75.8%	Rural nomadic	Eastern	25.8%	66.6%
Meru	Not marginalised	31.0%	Rural agrarian	Eastern	82.8%	78.3%
Nairobi^b^	Not marginalised	21.8%	Urban	Nairobi	89.1%	60.4%
Nyeri	Not marginalised	27.6%	Rural agrarian	Central	88.1%	77.8%
Turkana	Marginalised	87.5%	Rural nomadic	Rift Valley	22.8%	56.7%
Vihiga	Not marginalised	38.9%	Rural agrarian	Western	50.3%	90.9%
National average		45.2%			61.8%	67.5%

1. Kenya National Bureau of Statistics. Economic Survey. Nairobi, Kenya; 2014

2. Kenya National Bureau of Statistics, Ministry of Health, National AIDS Control Council, Kenya Medical Research Institute, National Council for Population and Development. Kenya Demographic and Health Survey: Key Indicators. Nairobi, Kenya; 2014

3. Counties considered marginalised are those which receive the additional equalisation fund for the fourteen most marginalised counties in the country

^a^Interviews also carried out with health workers from sub-county, health facility and community level

^b^ Interviews also carried out with health workers from sub-county, health facility and community level and interviews with CHVs, CHEWs, their supervisors and FGDs with community members

#### Analytical process

We adopted a framework approach to analysis in order to classify and organise data according to the key themes, concepts and emerging categories [24]. This included an inductive aspect, which allowed meaning to emerge from the data through familiarisation with the data by reading and re-reading through transcripts [23]. Following this a thematic framework was developed, which drew on understanding of the literature, the objectives of the interview, the themes within the data collection tool, experiences during field data collection and issues raised by the respondents themselves during interviews. NVivo 10 software was utilised to manage and code data. Following coding, data was charted to summarise findings while still retaining its context and essence, based on data from all ten counties and enabling analysis, according to Tanahashi’s themes: availability, affordability, acceptability, accessibility and quality of health services provided.

#### Quality assurance and ethical considerations

Qualitative data was recorded with participant’s consent and transcribed verbatim. Data collection continued until saturation was reached and data was triangulated between sources to minimise bias. Community and some health facility level respondents were interviewed by trained research assistants in Kiswahili or Kamba (depending on respondents’ preference). These interviews and discussions were translated to English, with a selection back-translated for quality checking. All participants were provided with information about the study and gave informed written consent. The research proposal was approved by Liverpool School of Tropical Medicine (Research Protocol 14.007 and Research Protocol 14.044) and Kenya Medical Research Institute (KEMRI) (Non-SSC Protocol 469). In addition, approval was received from the National Council for Science and Technology (NACOSTI) (NACOSTI/15/2058/4010).

## Results

Equity was widely discussed by all respondents, and was commonly considered to be a leading factor contributing to the need for devolution and one of the key values, which should underpin the priority-setting process. Improving equity was most commonly described in terms of improving access and bringing services closer to the ‘ordinary person’. County and national level respondents placed a strong emphasis on re-distribution of resources, based on geographic and financial access to health services in their understanding of equity. This is reflected in the study findings with much more extensive discussion surrounding availability and accessibility, the supply-side determinants, compared with demand-side determinants of coverage. However, one national level respondent highlighted this as a common weakness in the accepted understanding of equity within Kenya, with implications for demand for services.

*“For a very long time in fact we looked at equity from the lens of financial access and geographical access and a lot of the efforts were targeting that and ignoring other aspects …But actually there is quite number of barriers to access which we haven’t focused on and the policies have been very silent on that.”* National Respondent, Male11

In contrast to national and county level participants, respondents at sub-county and health facility level, placed a much stronger emphasis on quality, including the need for adequate drugs and staff to provide equitable and effective services. Community members, community health extension workers (CHEWs) and community health volunteers (CHVs) emphasised the need for a lack of prejudice, favouritism or tribalism, with services available for everyone. They also emphasised the importance of justice and their right to health care, along with quality, respectful and timely treatment at a location convenient to them.

*“[Fairness means] they should get high quality health services that reach everyone at the right time.”* CHV Team Leader, Female08

### Availability coverage

National policies were felt to influence equity and most respondents across all levels described improved equity between counties thanks to the equalisation fund for the most marginalised counties and consideration of poverty level within the ‘equitable share’. Yet upon reflection, we find that the national government’s medical equipment deal appears at odds with the stated emphasis on UHC, with some county governments not having the needed infrastructure or appropriately skilled staff to operate high technology equipment.

*“Instead of being given finances to come and budget they (county government) were forced to take some equipment, that’s what happened. You were to take equipment and you do not have the personnel who is able to run them and you were given. So they are lying all everywhere with no use… it’s not good by the way because like us we were given the CT scan machines and we have no personnel.”* County Non-Health Respondent, Female46

Respondents identified that after devolution there was increased funding available at the county level for health within formerly marginalised remote counties. Many county level respondents described investment to increase availability of services, infrastructure and allocation of staff for all health facilities, including the most remote.

*“I think under devolution, there is more equity, communities that were previously marginalized - I’m talking about Turkana, Mandera, Wajir, Garrisa, Moyale - are receiving unprecedented development, things that they never imagined they would get. There is also the equalization fund which is also meant for these historically marginalized areas which is helping them also.”* National Respondent, Male10

Investment in hospitals was a common priority for all counties, with nine out of ten county governments having either rehabilitated, upgraded or built a new county hospital. A minority of national respondents felt that investment in the county hospital may not have been the leading priority in terms of need within the county, but because it was a visible and tangible area for investment it was felt to have become a political priority.

*“You know when you go to a county, and you find that county leadership decides okay we want a nice gate to our county hospital, and then you walk into the county hospital and there are no drugs. Then probably the workers have not been paid. What does that say? It’s because the, the leader wants to say ‘you see we know our hospital is shining’. So it may not necessarily be speaking to the needs.”* National Respondent, Male07

In many cases expansion of infrastructure appears to have been entirely appropriate, particularly in formerly marginalised areas where there was a huge deficit and extremely limited geographic access to services. Many counties described investing in infrastructure for primary care, such as dispensaries and health centres which typically benefit poor populations more than rich. With county governments using their local knowledge of underserved areas to make more informed decisions regarding the location of new facilities.

Changing power structures since devolution were felt to have led to increased power held by politicians, rather than technical decision makers and health workers within the county. One of the challenges of this is the emphasis on visible curative services, rather than the less tangible (but essential) preventive services. Powerful political leaders were felt to accumulate more services, compared with less powerful leaders, regardless of need. Technical county level respondents at times felt that this had a negative impact on the level of equity within the county.

In many counties there were concerns raised, primarily by technical health decision-makers, that many of the health facilities constructed with the intention of improving availability of health services had not yet been staffed, equipped or added to the register to receive government drugs, supplies and funding. As a result, they remained non-functional and unable to provide effective health services to the population they were intended to benefit. A further threat looming, was seen to be the lack of future planning for these new facilities, with many health workers and health technical decision-makers across the counties highlighting that politicians and community simply do not appreciate the recurrent and maintenance costs needed for a health facility, potentially impeding functionality or quality of services provided and undermining future sustainability.

Community health services were widely acknowledged to expand availability, accessibility, acceptability and contact coverage of services and to perform a vital function in the quest for UHC (see acceptability and contact coverage sections). In around half of counties studied, there has been expansion in the availability of community health services since devolution. Whether community health was prioritised (or not) was felt to be related to the level of understanding about community health and political preference [8]. However, even where community units had been established there were, at times, coverage gaps. Respondents from one nomadic county in Northern Kenya, identified that prior to devolution CHVs had been identified during a community meeting held in the main town, with assumptions that each catchment village would have sent a representative. However, due to distance and inadequate mobilisation there were few attendees from the more remote catchment populations, with the result that CHVs were recruited predominantly from the main town. In order to ensure coverage of community health services in all villages, the county team were now returning to repeat the process. Respondents described innovation through recruitment of CHVs from within nomadic communities, who would therefore move with their community, even across country land borders.

*“So today you will find this group living in Kenya, tomorrow they have crossed the border to Uganda…, but you know you cannot find facility, health facility remains home. So we make sure that we get CHVs from those communities, so they move with these communities.”* County Health Respondent, Male39

Lack of variation in the number of CHVs for low density areas or hilly terrain created challenges for the CHVs, particularly those in pastoralist and some agrarian counties, with some CHVs having to travel up to 20 km between homesteads. As a result, CHVs and community members agreed that CHVs did not visit homes which were far away as frequently as homes close to their own, due to the long distance and lack of transport. Respondents from two counties described having introduced a modified community health structure, which accommodated varied population densities and terrain to address these challenges.

### Accessibility coverage

In addition to county government efforts to promote availability of services, national government have introduced several policies to increase access to services by increasing affordability of health services. These include the national policy for free maternal health care and removal of user fees from dispensaries and health centres, both introduced in 2013 shortly after introduction of devolution reforms, which was felt to have led to increased use of services:

*“Following the free medical services; the work load has grown very, very high.”* Health Worker, Male11

There were challenges with implementation of the free healthcare initiative however, due to lack of drugs available. This frequently resulted in patients who make contact with the health services still needing to pay in order to receive effective treatment, leading to the most poor remaining unable to receive effective care (see effective care section).

*“Every area you go to, you are told drugs are not here [government health facility]. They prescribe and you go buy outside. This has made the cost of treatment higher and not affordable …Of course that tells you that only the people with resources will now be able to access services that are relevant. People who are wealthy will afford to pay in the private clinics. People who are poor will wait and seek alternatives, like going for traditional medicine or self-treatment, self-medication on the counter.”* National Respondent, Male04

Improving geographic access to health services was described as an important priority for many counties (see availability section). The challenges of geographic access to health facilities were discussed in all counties, most extensively in those counties with nomadic populations. For those who lived far from the health facility (either in urban or rural areas) poor road infrastructure or seasonal weather changes created a barrier to accessing services. In these communities, outreach services formed a vital part of routine service delivery.

*“The county they should be ready to fund for an outreach because some places … when it rains it’s like they have been cut off in terms of transport.”* County Health Worker, Male03

Outreaches were identified by county, sub-county and health facility respondents as a means of improving health service coverage for the most remote communities, who did not have easy access to receive these services at a static health facility. The most widely described outreach services introduced since devolution, were the ‘beyond zero’ mobile clinics provided to each county by the First Lady’s Beyond Zero campaign to reduce maternal and neonatal mortality. These mobile clinics were felt to have contributed towards improved service access. However, there were still challenges surrounding their distribution, with all counties receiving one large bulky mobile clinic to achieve equality in distribution, regardless of level of need/ population size/ geography/ road terrain etc. As a result, people who live in the most remote and hard-to-reach areas remained unable to benefit, due to inadequate road infrastructure. Other forms of outreach services discussed included (donor) partner supported services, which typically remained unfunded by county governments following partner exit. Again, citizens living in the most remote areas remained unserved.

*“We normally have integrated outreach … we expect the County government to be filling those gaps and it is not forth coming so we have a very big problem. So we can say there are totally no access.”* Sub-County Respondent, Male04

### Acceptability coverage

Devolution provides counties with an opportunity to find new ways to stimulate demand for health services, e.g. through CHVs promoting use of health services, and to address barriers to the acceptability of services for patients with unique needs, e.g. deaf patients. This has at times been neglected, with a national member of the community health and development unit reporting that only half of counties have invested county government funds in community health services. Despite these planning deficits, CHVs (where present) were commonly described by facility and community level respondents as playing a key role in increasing acceptability and use of services.

*“The community is well mobilized … and you can see the number of deliveries has gone up. Our people don’t like coming to the hospital to deliver, but because this CHV is impacting, now they are able to come to the facility.”* County Health Respondent, Female40

In many counties, respondents described ensuring that a ramp is available at any new health facility which is being constructed, to ensure access for wheelchair users who reach the health facility (although reaching the health facility was recognised as a challenge for people with disabilities). Respondents from a few counties identified that CHVs were an important means of identifying and assisting people with disabilities and ensuring their linkage to support services.

“*They [CHVs] are able to reach out to them, in fact we are able to get some out especially the disabled, the children with disability that used to be hidden, and nowadays we can see them being brought forward*.” Sub-County Respondent, Female10

Having health workers trained in sign language at primary health facilities was an identified gap. Two counties had started or were planning to train health workers in basic sign language, to improve access to services for deaf patients.

### Contact coverage

Many counties have invested in hospitals and ambulances in efforts to strengthen referral services, but we found limited evidence to suggest this increased equitable contact with services (particularly for non-maternal health-related emergencies). Patients still need to pay user fees for services provided at hospitals (excluding maternal health services). Although waiver schemes are in place in many counties, challenges with long delays in reimbursement and limited scope for the payment (covers hospital fees only, rather than transport and other opportunity costs) continue to create barriers to patients making contact to use these services. Referral costs varied between counties, with no consistent policy regarding payment for ambulance referral. In one county, ambulance services were available free of charge for maternal health related emergencies. However, any other emergency could not use the ambulance, as a result of which the patient and their family would be obliged to seek private transport to reach a hospital. The major hospitals which offered a breadth of services and more experienced health workers were typically centred in urban areas, rather than the more remote places. Those living in more remote areas, who were poor were felt to experience a double challenge in reaching secondary level care.

By contrast to the challenges experienced by the most poor or those living in remote places with accessing level three services provided at the hospital, community members generally felt that community health volunteers (CHVs) (where present) improve contact with health services at the household level and prioritise attending the homes of those who are ‘vulnerable’. CHVs were often described by community members and their supervisors as providing additional support for their most vulnerable neighbours out of their own pockets.

In contrast to this norm, a small minority of community members in the most remote areas, felt that the CHV prioritises providing services to those in the community who are richer – those with a ‘pot belly’ or who have a tin roofed house, because “*he (CHV) will go to that person because he will get something there*.” Male community FGD02. However, the majority of community members, even in remote areas agreed with the dominant view described above, that CHVs prioritise visiting those who are disadvantaged.

### Effective coverage

There was limited emphasis on building the quality of health services provided following devolution. A range of respondents highlighted the inequitable dual level system for health (which pre-dates devolution). Under this system those who are rich pay for quality private care and those who are poorer receive perceived lower quality government services. Health workers and community respondents raised concerns around the quality of services provided at government facilities as a result of lack of drugs and supplies, particularly following increased patient contact coverage with services since removal of user fees at dispensaries and health centres.

Other quality concerns include insufficient numbers of staff, who are overworked. This was felt to lead to poor staff attitude as a result of stress and demotivation; clinician error due to tiredness and lack of support; long patient waiting times, with health workers having to serve up to 200 patients per day without the needed resources and support, a prerequisite to providing quality services.

*“I have a problem with the services the doctors give and I feel bad about it, because I came here one day and I was misdiagnosed. The quality of services in this health centre should be upgraded*.” Male community FGD04

## Discussion

Our findings reveal that devolution has brought wide ranging implications for health equity, some positive such as the inclusion of poverty within the equitable share of funds received by counties from national level, with support for formerly marginalised areas. Other positive findings include: increased availability of primary health facilities, typically in formerly underserved areas; efforts in some counties to promote acceptability of health services among deaf patients by training health workers in sign language; improved accessibility to services at household level, particularly for those most marginalised, through CHV home visits. However, alongside these positive findings there are also negative implications emerging since devolution with heavy investment in hospital equipment and infrastructure, which many of the most poor patients continue to struggle to access and use. In addition, political wrangling within some counties was perceived to influence decisions [8]. Further, lack of emphasis on quality, has in some cases undermined the provision of services, with newly constructed facilities remaining unfinished and therefore unable to provide effective services. Uneven investment in community health between counties, has led to varied scope for households to benefit from these services.

### Influence of devolution on the supply side

Devolution has brought improvements for the supply side, by expanding the availability and geographic accessibility of health services across many counties. These improvements have been undermined to some extent by heavy investment in improving the availability of hospital services, which predominantly benefit the rich [25], compared with community health services, which promote access to services among those considered marginalised [26]. In addition, insufficient emphasis to ensure that the required human resources and drugs and commodities accompany infrastructure, hinders the quality of these services. The medical equipment deal appears to be at odds with devolution, which specifies that county governments are responsible for the provision of services from level one to level three (and which therefore includes decisions about procurement (or not) of diagnostic equipment for use within county referral hospitals at level three) [27]. It is also working against the recommendations for UHC, which ought first to prioritise those high priority services which are of benefit to all citizens, before contemplating low priority ones, which benefit a smaller minority of citizens [16]. In the push to address geographic access, many counties have sought to build new health facilities and extend curative services, but public health and population measures such as promotive, preventive and rehabilitative services at community level, which are all necessary for universal health coverage have been neglected to varying degrees [15, 16]. This has previously been described in Indonesia, where public health services reportedly deteriorated following devolution, with reduced access among poorer populations [28, 29].

Investment in infrastructure and equipment have been focused across both primary health facilities and hospitals. While primary health care has previously been demonstrated to be pro-poor, public hospitals in Kenya have primarily been used by the rich, with the richest quintile benefiting from two thirds of all hospital outpatient services [25]. Hospitals can quickly absorb vast amounts of money. In Kenya they have previously consumed 50% of the health budget [25]. It is therefore crucial that hospital construction and refurbishment which will primarily benefit the rich, does not undermine community-based primary health care services which can benefit all. Emphasis on infrastructure over quality, as perceived by users, was previously demonstrated following decentralisation in Tanzania and Indonesia [30, 31]. There local leadership were poorly informed about health, lacking the understanding to recognise the benefits of public health services [31].

The combined effect of devolution and abolishing of user fees have implications for equity in maternal health care, where demand has increased, but quality has not and neither has awareness of entitlement. This creates a tension in different country contexts. Our findings reveal that removal of user fees increased use of health services, in keeping with previous study in Kenya [32]. Gitobu et al. (2018) revealed that the number of deliveries in health facilities increased by 29.5% following implementation of the free maternal health services policy remaining consistent over the two year period following introduction of the policy [32]. In keeping with findings from our study, Gitobu et al. (2018) highlighted concerns about diversion of the free maternal health care funds by county governments, with implications for the quality of services [32].

Continued user fees (for non- maternal health services) at hospitals, with lengthy waiver process which did not cover opportunity costs was felt to contribute towards the continued exclusion of the most poor from receiving these services. Frequent supply chain gaps meant that health service users (who should receive free services) still needed to buy drugs elsewhere as described previously in Kenya, following introduction of devolution reforms [33, 34], resulting in continued exclusion of the most poor from effective services. Although recent study has indicated that when counties managed to procure drugs, health facilities reported a better order fill-rate, compared with prior to devolution [35]. The introduction of these policies at the same time as devolution has previously been recognised to have influenced their implementation, leading to compromised quality by operational challenges including delayed reimbursements at health facilities and exacerbation of existing weakness, including shortages of health workers and drugs and supplies [33]. There have been many discussions about increasing enrolment in national health insurance fund (NHIF) in Kenya, as a pathway to improving access to health services. As a result the Kenyan government have extended the service package to include outpatient as well as inpatient services, and have introduced an NHIF subsidy programme to identify and provide subsidy for NHIF membership for the poorest households [13]. However, as our study highlights, in order for patients to receive effective health services, any intervention to increase insurance coverage, must have a strong quality emphasis, to ensure that services covered under NHIF enrolment are of good quality and provide effective coverage.

### Influence of devolution on the demand side

In order to attain universal health coverage, services must also be acceptable to the population if they are to be utilised. Demand-side barriers including cultural and religious barriers, decision-making and gender autonomy and access to knowledge and information about health and services must first be addressed and overcome if health services are to be used [36]. Similar to other countries, devolved counties in Kenya have generally been slow to approach these barriers [37]. As we have published elsewhere, a few counties in Kenya have introduced demand generation strategies, such as community health approaches to encourage appropriate use of health services. However, most counties have not yet addressed constraints to accessing services, such as the acceptability of skilled delivery through engaging with cultural and religious beliefs and different community perceptions of health workers [38]. Community health approaches can address and reduce many of these barriers [26], alleviating and reducing the forces which reinforce exclusion and thereby helping to improve acceptability and use of services. When CHWs are adequately empowered there is opportunity for them to act as community advocates [39], playing a key interface role in mediating demand side factors for equity and responding to the unique opportunities afforded them as a consequence of their intermediary position between the health system and the community [40].

Quality was rarely described as a value for priority-setting and infrequently described as a priority which counties were seeking to address. Instead, quality gaps such as limited functionality of community health services, lack of consistent drug supply chain (in some counties), lack of funds to support supervisors were described. As a result of the perceived lack of quality at public health facilities a ‘rich-poor’ divide was evident. While this is not a new phenomenon since devolution in Kenya, county governments have so far demonstrated little commitment to improving the quality of health services at public health facilities. In fact some of the interventions introduced prior to devolution to promote quality, such as transferring funds directly from the national treasury to health facility bank accounts [25], have potentially been undermined as a result of the control of funds at county treasury level, leading to delayed and lower transfers to health facility bank accounts. If not addressed, this may lead to a widening divide between rich and poor patients, as occurred in Indonesia with rich patients attending private health facilities and poor patients attending public health facilities, where the service quality was perceived to be poor [29]. Quality improvement need not be costly, rather approaches which empower local solutions to quality through dialogue, rewarding best practice and advocacy can bring quality improvements with a modest resource investment [41]. With regards to community health services, supervision and the policy environment can affect the quality of community health services provided [42].

### Use of the Tanahashi framework

**Fig. 1:**
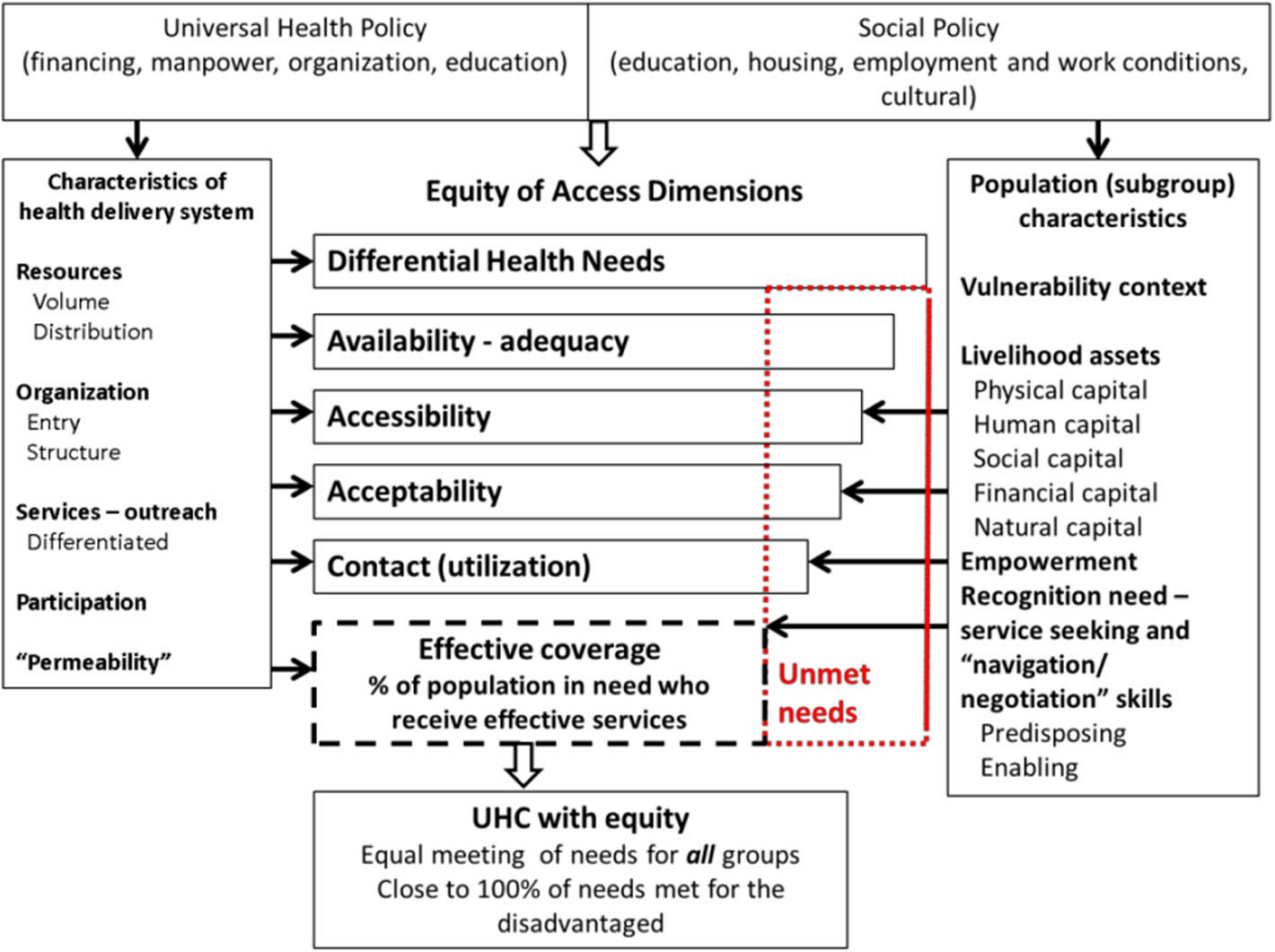
UHC and equity of access. Effective coverage for all health needs as a result of supply and demand ‘fit’. Source page 15, Frenz and Vega, 2010

Application of the Tanahashi model to analyse equity changes following devolution provided scope to consider the various aspects of coverage (availability, accessibility, acceptability, first contact, effective coverage) and to identify potential bottleneck areas which can impede effective coverage [19]. It also allows space to identify groups with unmet needs [43]. Our findings are in keeping with work by Frenz and Vega, which highlights the interaction between supply and demand at various levels and the need for concern for groups with greater needs (see Fig. 1). This framework highlights how health and social policy, can influence the characteristics of the health system and the degree of vulnerability, livelihoods, empowerment and recognition of need for health services, within the population [43]. Dramatic reforms such as devolution have the potential to create seismic change, through revision of sub-national priorities for health and social policy and implementation. We find that county governments are taking many positive actions to expand the supply of services and characteristics of the health system, by removing some of the geographic and financial barriers to utilising services. There is, however, still need for a greater emphasis on improving the demand-side, by addressing and providing services which are acceptable to the population. Likewise, there is need for greater recognition of the needs of people who are most marginalised. Intersectoral collaboration seeks to address vulnerabilities, livelihoods, empowerment and recognition of the need for services among communities experiencing greatest needs, e.g. people living in the most remote areas. Several Latin American countries have employed intersectoral approaches to improve participation and the degree to which people with differing experience of power can benefit from and use health services [44]. These approaches have shown some degree of success, contributing towards improving population health outcomes and addressing equity [44]. Kenya’s devolved county governments have opportunity to invest in tailor made health care services, which include promotion and prevention measures that address the social determinants of health, perhaps learning from Brazil’s community-based family health programme. Other opportunities include the ability to identify marginalised groups in collaboration with other county departments and to plan actions which can both increase their access and use of health services, but also change their social determinants. Learning lessons from programmes such as the “Bolsa Familia” in Brazil, which seeks to create employment opportunities and income transfers for families in poverty, along with increasing access to public services will be strategic [44].

We find value in using the model as a framework to consider qualitative findings surrounding the process and content of priorities from an equity perspective following the introduction of devolution.

## Conclusions

Devolution in Kenya has brought varied implications for health equity as outlined through the availability, accessibility, acceptability, contact with and quality of services provided. To date much of the focus has been on improving the availability, and accessibility of health services, which are helping to improve health equity for many. Yet if Kenya is to achieve universal health coverage for all citizens, then county governments will need to go further by ensuring that actions are introduced which increase acceptability, use and effective coverage of quality services. Community health services can play a crucial role to meet both the supply and demand-side aspects of health equity.

## Footnotes

1 Services should be grouped into priority classes using criteria such as cost-effectiveness, priority to the worse off and financial risk protection [16].

2 REACHOUT is an ambitious five year international research consortium aiming to generate knowledge to strengthen the performance of CHWs and other close-to-community (CTC) providers in promotional, preventive and curative primary health services in six low- and middle-income countries in rural and urban areas in Africa and Asia, including Kenya.
